# Prenatal diagnosis of chromosome 18 long arm deletion syndrome by high-throughput sequencing: Two case reports

**DOI:** 10.1097/MD.0000000000028143

**Published:** 2021-12-17

**Authors:** Xuechun Bai, Lianwen Zheng, Shuai Ma, Xun Kan

**Affiliations:** The Second Hospital of Jilin University, Changchun City, Jilin Province, China.

**Keywords:** amniocentesis, case report, chromosome 18, chromosome long arm deletion, high-throughput sequencing

## Abstract

**Rationale:**

Chromosome 18 long arm deletion syndrome is a group of clinical syndromes caused by partial or total genetic material deletion of the long arm of chromosome 18 (18q), whose clinical manifestations are related to presentation and developmental abnormalities in various aspects such as intelligence, face, and movement. Prenatal diagnosis of this syndrome is challenging because of its low incidence and uncharacteristic prenatal clinical performance. In this paper, 2 cases of partial deletion of 18q found in prenatal amniotic fluid examination by high-throughput sequencing were reported and analyzed.

**Patient concerns:**

In patient 1, non-invasive prenatal gene detection at 21 + 2 weeks of gestation suggests a risk of trisomy 18. In patient 2, ultrasound examination at 21 + 2 weeks of gestation revealed a single live fetus, but it was difficult to pinpoint whether the fetus had only 1 umbilical artery to supply blood.

**Diagnosis and intervention:**

The 18q deletion syndrome was diagnosed by chromosome karyotype analysis and high-throughput sequencing.

**Outcomes:**

The pregnancies were terminated due to the abnormal chromosome.

**Lesson:**

This report adds novel variants to the genetic profile of 18q deletion, in order to enrich the genetic data of long arm deletion of 18 chromosomes and provide better services for pre-screening, diagnosis, and genetic counseling for this disease.

## 1 Introduction

Chromosome 18 long arm deletion syndrome is a group of clinical syndromes caused by partial or total genetic material deletion on the long arm of chromosome 18 (18q), with a low incidence of approximately 1:40,000^[1]^ in neonates. Approximately 94% of children with 18q deletion are newly occurring chromosomal aberrations, and the other 6% are caused by unbalanced chromosomal translocation, as their parents are usually carriers of balanced chromosomal translocation.^[2]^ The phenotype of 18q deletion syndrome varies greatly due to the location of chromosome breakage, the type of missing genes, and individual characteristics. However, at present, it is clear that most structural or numerical malformations resulting from deletions always produce significant adverse phenotypes. Its clinical manifestations involve abnormal development of various systems, such as intelligence, face, movement, and so on.^[2–5]^

Screening for structural or numerical abnormalities of chromosomes is usually carried out at 11^+0^ to 13^+6^ weeks of gestation and is used as an indispensable project of fetal routine examination in the second trimester of pregnancy to detect suspicious patients by ultrasound measurements or by combined test of serological indicator in early and second trimester of pregnancy.^[6]^ Nevertheless, the results of these screenings are indirect methods that are affected by various factors such as gestational age, fetal posture, maternal body shape, amniotic fluid, and fetal movement. Therefore, it is difficult to exclude all fetal malformations and it has great uncertainty and inaccuracy, and this is why they can only be used as a simple screening method.^[7]^ The use of only 2 methods for the examination of fetal chromosomal abnormalities will induce a greater rate of missed diagnosis and misdiagnosis rates. On the other hand, in view of the serious consequences of 18 chromosome deletions in offspring, there is an urgent need for more accurate examination methods to determine whether there are chromosomal malformations in embryos to determine further diagnosis and treatment options.

As a new detection method for chromosomal abnormalities, high-throughput sequencing can not only detect aneuploid changes, but also ascertain microdeletions and microduplication of 100 kb to submicroscopic levels of DNA fragments on chromosomes by copy number variation (CNV) detection.^[8]^ It is characterized by massively parallel sequencing to dissect detailed genetic maps through tests covering the whole genome to obtain more accurate information on genetic changes.^[9]^ This more accurate pre-screening method can not only compensate for the shortcomings of traditional chromosome karyotype analysis, such as low resolution and inability to determine the source, size, and location of derived chromosome fragments, but also be a powerful supplement to chromosome karyotype analysis with higher accuracy and accuracy.^[10]^

In this study, after preliminary chromosome karyotype analysis of fetal free DNA obtained from amniocytes, high-throughput sequencing was used to further clarify the variation in fragment size, locus, and copy number of chromosomal deletions, in order to enrich the genetic data of 18q deletion and improve the screening, diagnosis, and genetic counseling of this disease.

## 2 Case introduction

This study was approved by the Institutional Review Board of Jilin University Second Hospital (ethical approval number: 2021-108) and followed the tenets of the Declaration of Helsinki. Informed consent was obtained from the patient for the publication of this case report and accompanying images.

### 2.1 Case 1

A 27-year-old G1P0 pregnant woman, whose results of non-invasive prenatal gene detection at 21^+2^ weeks of gestation, suggested that the risk of trisomy 18 (– 6.6) was high, while the risk of trisomy 21 (0.681) and trisomy 13 (2.363) was low. Ultrasound examination at 23^+2^ weeks of gestation revealed a single live fetus with a gestational age of 23^+2^ weeks in utero, whereas the umbilical cord was wrapped around the neck of the fetus for 1 week. The measurement indexes of fetal development are as follows: biparietal diameter = 6.0 cm, head circumference = 20.9 cm, abdominal circumference = 17.4 cm, femur long = 4.0 cm, amniotic fluid index = 16.7 cm, and the 3 values of umbilical artery S/D were 4.6, 3.2, and 2.7 respectively. It is suggested that amniocentesis, chromosome karyotype analysis, and CNVs should be recommended to further confirm the condition of the fetal chromosome.

**Figure 1:**
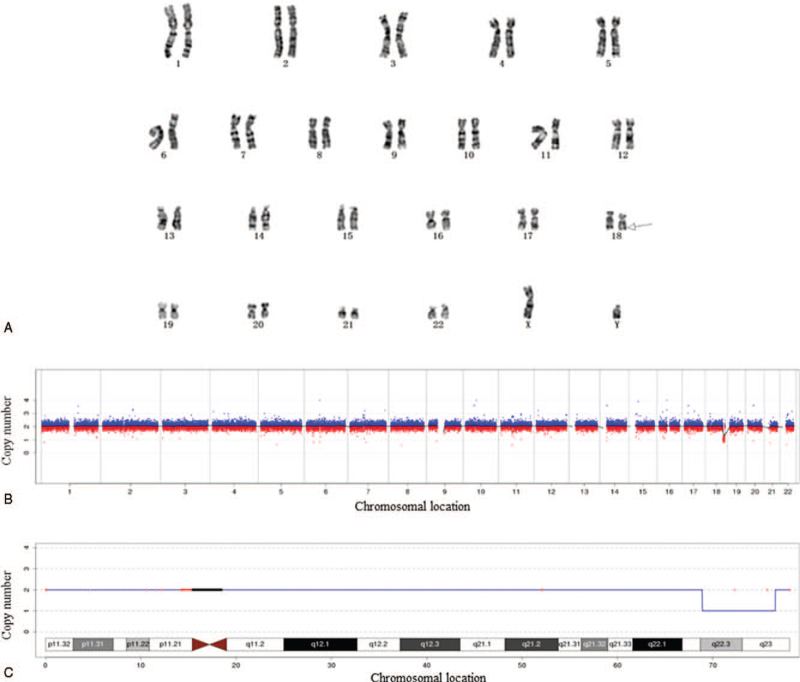
Genetic test results. Prenatal diagnosis of monosomy 18q deletion syndrome in Case 1. (A) Result of chromosome karyotype: The fetal chromosome karyotype was 46, XY, del (18) (q22.3; q23). The region indicated is the chromosomal deletion region of the patient. (B and C) Results of genetic testing: The description of genome-wide detection is 7.66 Mb region deleted at q22.3-q23 on chromosome 18. 18q = long arm of chromosome 18.

Chromosome karyotype analysis after amniocentesis showed that the fetal chromosome karyotype was 46, XY, del (18) (q22.3; q23) (Fig. 1A). CNV detection results showed that this sample detected chromosome aneuploidy or CNVs of more than 100 kb, which was regarded as the definite pathogenic genomic: 18q22.3q23 (68900001-76560000), and the size of the deleted fragment was 7.66 Mb (Fig. 1B, C).

### 2.2 Case 2

A 35-year-old G4P1 pregnant woman who had 1 natural birth and 2 induced abortions. The results of non-invasive prenatal gene detection at 19^+2^ weeks of gestation suggested that the risk of trisomy 21 (–1.249), trisomy 18 (–1.534), and trisomy 13 (–1.904) were all at low levels. Ultrasound examination at 21^+2^ weeks of gestation revealed that there is a single live fetus with a gestational age of 21^+2^ weeks in utero, whereas it is difficult to determine whether the fetus has only 1 umbilical artery to supply blood. The measurement indexes of fetal development are as follows: biparietal diameter = 4.9 cm, femur long = 3.6 cm, and the deepest amniotic fluid was 5.7 cm. The couple requested amniocentesis, chromosome karyotype analysis, and CNVs to further confirm the diagnosis.

**Figure 2:**
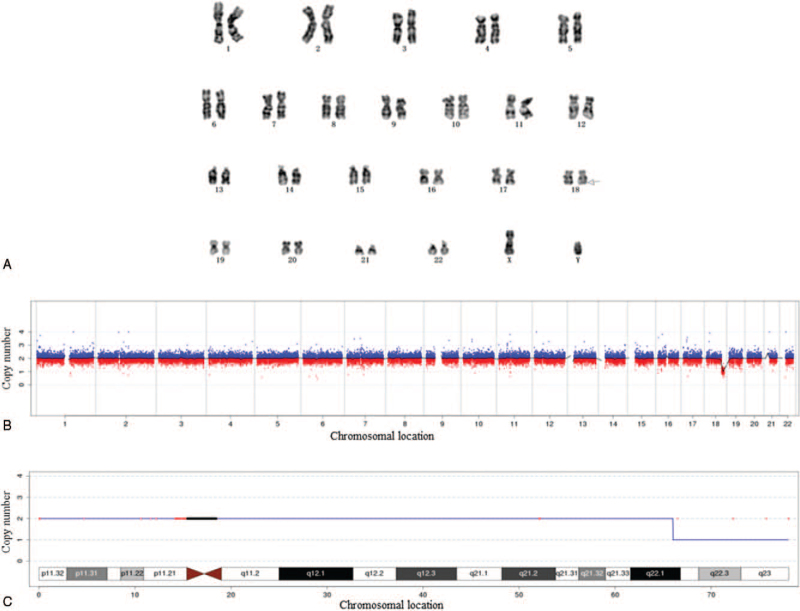
Genetic test results. Prenatal diagnosis of monosomy 18q deletion syndrome in Case 2. (A) Result of chromosome karyotype: The fetal chromosome karyotype was 46, XY, del (18) (q22.2; q23). The region indicated is the chromosomal deletion region of the patient. (B and C) Result of genetic testing: Description of genome-wide detection is 12 Mb region was deleted at q22.1-q23 on chromosome 18. 18q = long arm of chromosome 18.

Chromosome karyotype analysis after amniocentesis showed that the fetal chromosome karyotype was 46, XY, del (18) (q22.2; q23) (Fig. 2A). CNV detection results showed that this sample detected chromosome aneuploidy or CNVs of more than 100 kb, which was regarded as the definite pathogenic genomic: seq [hg19] del (18) (q22.1q23), chr18: g.6602000178020000del (Fig. 2B, C).

After half a year, the personnel of the prenatal diagnosis center followed up the 2 couples and learned that both couples voluntarily terminated their pregnancy after knowing the results of amniocentesis.

## 3 Discussion

**Figure 3:**
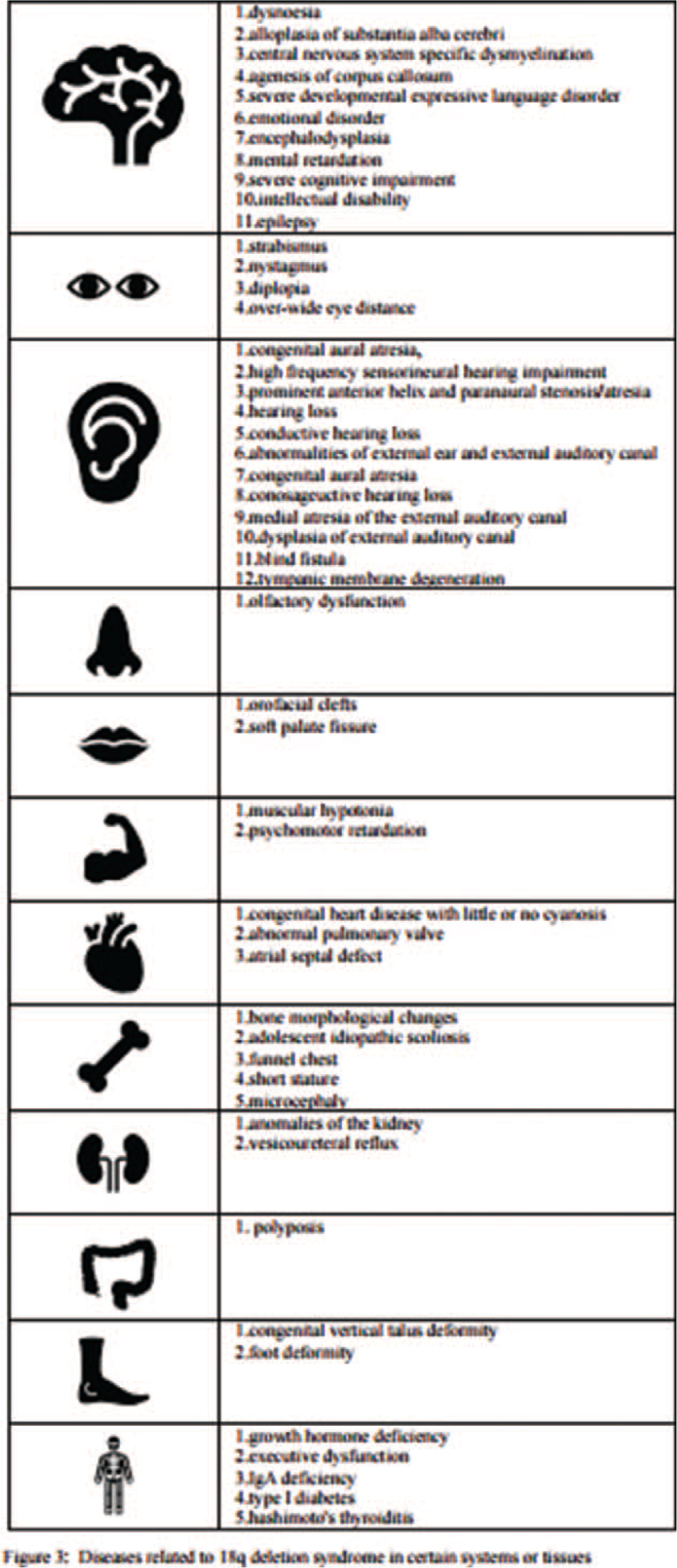
Diseases related to 18q deletion syndrome in certain systems or tissues. This figure enumerates the currently reported diseases or clinical manifestations of 18q deletion syndrome. 18q = long arm of chromosome 18.

18q deletion syndrome is a group of heterogeneous diseases caused by the deletion of all or part of the 18q, whose clinical manifestations involve abnormal development of various systems such as intelligence, face, and movement. It frequently occurs randomly for unknown reasons, while most cases result from terminal deletion of 18q, and a few cases with monosomy 18q deletion syndrome were reported as a result of an unbalanced arm translocation. Various clinical phenotypes have been reported at present (Fig. 3).^[11,12]^ One patient with 18q22.3-q23 deletion had major clinical characteristics characterized by psychomotor retardation, language development delay, moderate intellectual disability, hearing loss, epilepsy, electroencephalographic abnormalities, behavioral abnormalities, special face.^[13]^ The main clinical features of a patient with 18q22.3-q23 deletion were short stature, delayed growth, linguistic retardation, conductive hearing loss, abnormalities of the external ear, external auditory canal, and soft palate fissure.^[14]^ Three members of another family carried the 18q22.3-q23 deletion fragment, and congenital vertical talus, bilateral hearing loss, and special face are their common clinical features.^[15]^ To our knowledge, none of the previously reported interstitial microdeletions involving 18q22.3-q23 or 18q22.2-q23 overlap with the cases described in our report, and the pathogenicity of CNVs with this deletion has not been previously described. However, it is a pity that the special performance of fetal in the 2 cases mentioned above is not obtained because the parents have not chosen our hospital to terminate pregnancy.

**Figure 4:**
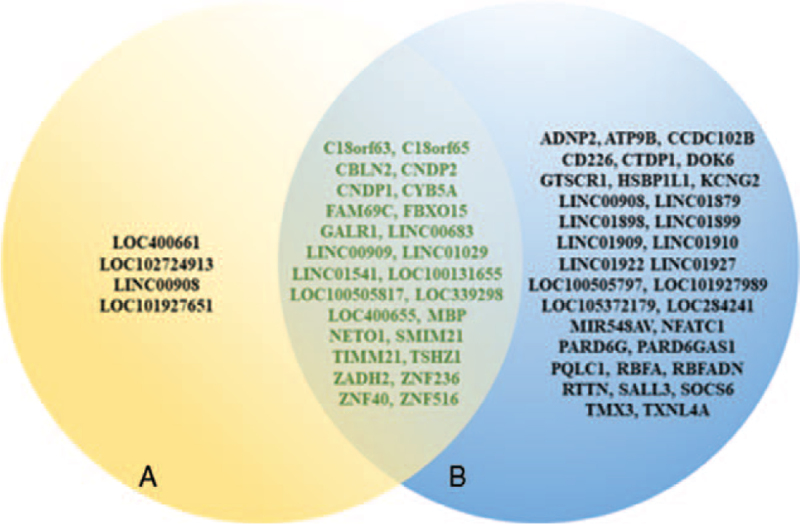
Genes involved in the case. Shown in the figure are the genes involved in the 2 cases in this paper, and the intersecting parts of the 2 circles are the genes involved in common. (A) Genes involved in Case 1. (B) Genes involved in case 2.

Chromosomal deletions are known to lead to abnormal expression of relevant deleted genes, thus displaying a wide variety of clinical phenotypes. Through the UCSC Genome Browser on Human and current published literature,^[16,17]^ the genes missing from the 2 cases in this paper can be discovered (Fig. 4). There is a special gene widely reported among the deleted region contained in all cases above, named TSHZ1, which can lead to congenital aural atresia if the haploinsufficiency of this gene is insufficient.^[18]^ Congenital aural atresia is an autosomal dominant genetic disease with a string of main clinical manifestations, such as bilateral congenital hearing loss, medial atresia of the external auditory canal, dysplasia of the external auditory canal, blind fistula, tympanic membrane degeneration, and olfactory dysfunction.^[13]^ When the deletion gene includes *MBP*, in addition to the common symptoms of 18q deletion syndrome such as microcephaly and dysaudia, there are also some autoimmune diseases, including type I diabetes and Hashimoto's thyroiditis.^[19]^ Thereafter, some studies have reported that the key regions encoding white matter diseases include 5 genes: ZNF516, ZNF236, LOC284276, MBP, and GALR. Short stature or growth hormone deficiency involves the GALR1 gene, which encodes a growth stimulating hormone neuropeptide that stimulates secretion of growth hormone.^[20]^ Patients with IgA deficiency and immune abnormalities are susceptible to respiratory/digestive system infectious diseases. The coding gene for IgA deficiency maps to the 18q22.3q23 region, approximately 7 Mb in size. This region contains many genes, of which the NFATC1 gene is especially noteworthy, which encodes activated T cells that are expressed in the endocardium and plays an important role in the formation of heart valves. Mice have severe valvular heart diseases, such as abnormal pulmonary valves and atrial septal defects, when this gene is deleted.^[20,21]^ Unfortunately, there is still a large proportion of genes whose role is not clear in the pathogenesis of 18q deletion and requires further investigation.

Furthermore, what is noteworthy is that it is difficult to identify these features by ultrasound measurements or by combined test of serological indicator in early and second trimester of pregnancy. Therefore, accurate prenatal diagnosis is urgently needed in cases with 18q deletion syndrome. At present, high-throughput sequencing can be used to identify underlying gene mutations if an early test is positive for a condition. Whether or not to become an inflection point in the process of desert biochemical screening and begin with high-throughput sequencing of genes or whole-exome or genome sequencing is a hot topic of debate in the medical field as high-throughput sequencing continues to become faster and cheaper.^[22,23]^

## 4 Conclusions and perspectives

There are great differences in the clinical phenotypes caused by deletions at different sites on chromosome 18. Moreover, it often presents with mental retardation and related diseases in different age groups. At present, there is almost no treatment to cure such diseases; nevertheless, timely and accurate prenatal diagnosis is particularly important. To date, various techniques for prenatal diagnosis are in the stage of vigorous development, but it is a pity that deletions at different sites on chromosome 18 cannot be accurately detected. However, with the development of high-throughput sequencing technology, more detailed gene deletion information has been obtained. Therefore, we hope that the report of 2 cases of 18q deletions in this paper can help us to better establish a clinical database of chromosome 18 deletions, in order to provide some experience for prenatal diagnosis and genetic counseling of chromosome 18 deletion syndrome.

## Acknowledgments

The authors thank all 3 probands and their families for their cooperation. The authors thank the laboratory for their expert laboratory work and analyses.

## Author contributions

Clinical consultation: Wang Yuxia.

Data collection: Wang Min.

**Data curation:** Xuechun Bai, Shuai Ma.

**Supervision:** Lianwen Zheng, Xun Kan.

**Writing – original draft:** Xuechun Bai.

**Writing – review & editing:** Xuechun Bai, Lianwen Zheng, Xun Kan.
